# Role of gut microbiota in doxorubicin-induced cardiotoxicity: from pathogenesis to related interventions

**DOI:** 10.1186/s12967-024-05232-5

**Published:** 2024-05-08

**Authors:** Chao Huang, Xiaoxia Li, Hanqing Li, Ruolan Chen, Zhaoqing Li, Daisong Li, Xiaojian Xu, Guoliang Zhang, Luning Qin, Bing Li, Xian-Ming Chu

**Affiliations:** 1 Department of Cardiology, The Affiliated Hospital of Qingdao University, No. 59 Haier Road, Qingdao, Shandong 266100 China; 2 Department of Genetics and Cell Biology, Basic Medical College, Qingdao University, No. 308 Ningxia Road, Qingdao, Shandong 266000 China; 3 Department of Gastroenterology, The Affiliated Hospital of Qingdao University, No. 16 Jiangsu Road, Qingdao, 266000 China; 4 Department of Dermatology, The Affiliated Haici Hospital of Qingdao University, Qingdao, 266033 China; 5 The Affiliated Cardiovascular Hospital of Qingdao University, No. 5 Zhiquan Road, Qingdao, 266071 China

**Keywords:** Cardiotoxicity, Doxorubicin, Gut microbiota, Metabolites, Interventions, Pathogenesis

## Abstract

Doxorubicin (DOX) is a broad-spectrum and highly efficient anticancer agent, but its clinical implication is limited by lethal cardiotoxicity. Growing evidences have shown that alterations in intestinal microbial composition and function, namely dysbiosis, are closely linked to the progression of DOX-induced cardiotoxicity (DIC) through regulating the gut-microbiota-heart (GMH) axis. The role of gut microbiota and its metabolites in DIC, however, is largely unelucidated. Our review will focus on the potential mechanism between gut microbiota dysbiosis and DIC, so as to provide novel insights into the pathophysiology of DIC. Furthermore, we summarize the underlying interventions of microbial-targeted therapeutics in DIC, encompassing dietary interventions, fecal microbiota transplantation (FMT), probiotics, antibiotics, and natural phytochemicals. Given the emergence of microbial investigation in DIC, finally we aim to point out a novel direction for future research and clinical intervention of DIC, which may be helpful for the DIC patients.

## Introduction

   With the significant advancements in cancer treatment and detection technologies, the survival rate of cancer patients has greatly improved in the past half-century [1, 2]. Doxorubicin (DOX), an efficient anthracycline antibiotic, has become a cornerstone in the chemotherapy of various cancers, such as breast cancer, lymphoma, and leukemia [3]. However, approximately a quarter of the patients who receive this highly efficient treatment ultimately discontinue their regimen due to the potential DOX-induced cardiotoxicity (DIC), which greatly hampered the clinical application of DOX [4]. Despite the DIC has attracted widespread clinical attentions, clinically available drugs for the treatment of this condition are limited [5]. In recent years, mounting studies have verified the probable molecular mechanisms underlying DIC, and developed cardioprotective adjuvants for potential targets, such as antioxidants [6], AMP-activated protein kinase (AMPK) agonists [7, 8] and other natural compounds [9]. However, none of these strategies is unanimously recommended and many drug researches lack clinical data, emphasizing the need for further studies [10].

   Recent evidences indicated that gut microbiota have been closely linked to multiple cardiovascular diseases through regulating “gut-microbiota-heart axis” [11–14]. It has been found that metabolites produced by the gut microbiota, short-chain fatty acids (SCFAs), are not only associated with energy metabolism [15] but also involved in the development of DIC [16–18]. Importantly, a recent in vivo study demonstrated for the first time that exogenous supplementation of phenylalanine-butyramide (FAB) protected against DIC by preventing mitochondrial dysfunction [18]. In this review, we will concentrate on an array of studies investigating the role of gut microbiota in alleviating DIC. We will emphasize the supporting evidence for the cardioprotective effects of both gut microbiota and their metabolites. Finally, we summarize the potential interventions of microbiota-targeted therapeutics in DIC. We aim to offer novel perspectives that might contribute to developing potential therapeutic strategies to prevent DIC.

## The molecular mechanism of DIC

   Multiple mechanisms have been proposed in DIC, including oxidative stress [19], mitochondrial dysfunction [20], autophagy [21], calcium dysregulation [22], cardiac metabolic alterations [23] and regulated cell death [24]. Nevertheless, the precise mechanism of DIC remains incompletely understood and subjects to debate. The prevailing belief among scholars is that inflammation plays a pivotal role in the development of DOX-induced cardiomyopathy [25, 26]. The nucleotide-binding domain-like receptor protein 3 (NLRP3) inflammasome is as significant part of the innate immune system. It can be activated by toll-like receptors (TLRs), nuclear factor-κB (NF-κB), reactive oxygen species (ROS) and other signals [27]. DOX can also activate NLRP3 inflammasome. It was reported to stimulate the production of caspase-1 and the release of proinflammatory cytokines, such as IL-1 β and IL-18, which caused cardiomyocyte death and inflammatory response in cardiac tissue [28, 29]. Another in vivo study revealed that vitamin C improved cardiac structure and function in DIC by reducing oxidative/nitrosative stress, apoptosis, and inflammation. They found DOX could increase the secretion and release of NF-κB, which leads to inflammation reaction [30].

**Fig. 1:**
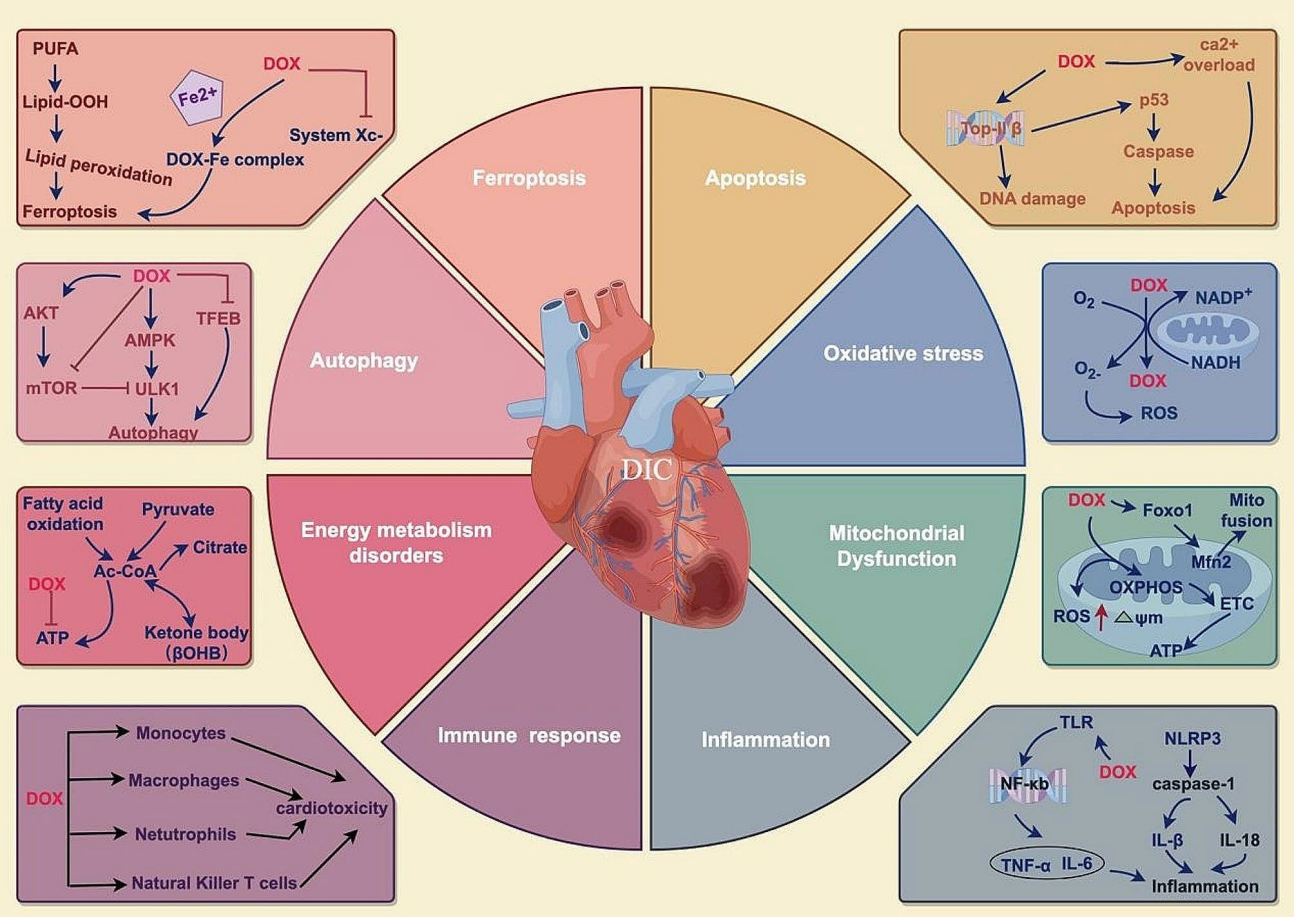
Simplified scheme of the molecular mechanism on doxorubicin-induced cardiotoxicity. Oxidative stress and inflammation are considered to be the main molecular mechanisms of DIC. Furthermore, programmed cell death, including apoptosis and ferroptosis, has been revealed to be involved in DIC. DIC is also associated with mitochondrial dynamics abnormalities, which plays an important role in the regulation of cardiac energy balance during physiological or pathological stress, as well as changes in mitochondrial quality control [42]. *Abbreviations* ATP, adenosine triphosphate; AMPK, AMP-activated protein kinase; AKT, protein kinase B; Ac-CoA, acetyl-coenzyme A; DOX, doxorubicin; ROS, reactive oxygen species; OXPHOS, oxidative phosphorylation; PUFA, polyunsaturated fatty acids; System Xc-, cystine-glutamate antiporter; TFEB, transcription factor EB; TLR, toll-like receptors; NLRP3, NOD-like receptor 3; NADPH, nicotinamide adenine dinucleotide phosphate; The figure was created with Figdraw (https://www.figdraw.com/)

   It is notable that the immune cells play a dual role in cardiac homeostasis and disease [31]. On the one hand, a large number of immune cells are recruited to the heart to remove dying tissue and promote healing after myocardial infarction; on the other hand, they can also cause irreversible damage under some circumstances, which contributes to heart failure. Interestingly, recent studies have shown that immune cells and cytokines are involved in DOX-induced heart injury via activating inflammation [32]. For instance, a study has shown that monocytes and macrophage apoptosis is linked to acute inflammation brought by DOX [33]. Mechanistically, the TLR2/TLR9-MyD88 pathway mediated the innate immune’s perception of apoptotic cells, which was essential for the start of an acute inflammatory response to DOX. Although investigations have pointed out the importance of inflammation in causing cardiomyopathy, it is increasingly clear that DOX cardiotoxicity is triggered by multiple complex mechanisms. The mainstream established pathomechanisms of DIC are briefly summarized in Fig. 1, such as oxidative stress [6], ferroptosis [34], apoptosis [35], autophagy [36–38], and energy metabolism disorders [39].

   With the growing understanding of DIC, the intervention of inflammation [40] and autophagy [41], are constantly being updated. Recent advances in molecular biology have significantly expanded the scope of research on DOX cardiotoxicity.

## Gut microbiota and DOX-induced cardiotoxicity

### Brief overview of gut microbiota

   Trillions of microorganisms inhabit in human guts, including bacteria, viruses, archaea and fungi [43, 44]. The caecum and proximal colon are the regions with the highest concentration of microbial biomass in the digestive system [45]. Extensive studies show that gut microbiota is crucial for the fermentation of dietary fibers and the synthesis of vitamins. Furthermore, they also exert a vital role in maintaining intestinal health and regulating the immune system [46]. Notably, the *Bacteroidetes*(B) and *Firmicutes*(F) are two dominant bacterial phyla in the healthy gut. A decrease in the F/B ratio is generally associated with a healthy gut microbiome, while a higher ratio may indicate a disrupted microbial balance, potentially related to certain diseases or health conditions [47]. A low F/B ratio is frequently attributed to a reduction in certain beneficial bacteria, which are subsequently replaced by potentially harmful bacteria within the same phyla, particularly *Firmicutes*. This alteration of the F/B ratio influenced by distinct lifestyle factors, such as dietary choices, sleep patterns, physical activity and medication usage.

### Gut microbiota and DIC risk factors

   The connection between gut microbiota and cardiovascular risk factors, like obesity, advanced age, and hypertension, is complex and multifaceted. These risk factors can affect systemic conditions, including metabolic changes, chronic inflammation, and gut microbiota dysbiosis [48]. In turn, intestinal microbiota dysbiosis can lead to the worsening of these risk factors [49, 50]. For example, the progression of diabetes has been associated with a decrease in the abundance of certain bacterial groups, including *Firmicutes* and *Bacteroidetes* [51]. Similarly, studies have shown decline in the ratio of bacteria produced butyrate in individuals with diabetes [52]. Furthermore, multiple bacterial species were found to be linked to hypertension, including *Lactobacillus*, *Klebsiella*, *Parabacteroides*, *Desulfovibrio*, and *Prevotella* [53, 54]. Previous evidence showed that *Lactobacillus* could reduce hypertension involving in modulation of sympathetic activity, oxidative stress, vascular tone, and inflammatory response [55].

   Similarly, advanced age is linked to alterations in the composition and abundance of gut microbiota [56]. Older adults tend to have lower levels of core commensal bacteria like *Bacteroides*, *Bifidobacterium*, and *Firmicutes*, which can contribute to health deterioration [57, 58]. Additionally, cancer patients often experience gut microbiota dysbiosis, which can further complicate their cardiovascular risk factors [59].

   Notably, there is evidence suggesting a connection between cardiovascular risk factors and DIC [60]. For example, research findings indicated that there exists an elevated likelihood of cardiac dysfunction in individuals aged 65 years and above, as opposed to their younger counterparts [61]. Although there is evidence linking gut microbiota, DIC, and cardiovascular risk factors, further investigation is required to completely comprehend this interaction. Therefore, understanding the link between intestinal flora and cardiovascular risk factors and restoring gut microbiota dysbiosis in cancer patients before doxorubicin treatment could be a promising strategy to reduce DIC.

### Gut-microbiota-heart (GMH) axis

   As raised above, the communication between the gut microbiota and the cardiovascular system is bidirectional [62]. This is because extensive research views the gut microbiota as a “endocrine organ” [63, 64]. On one hand, cardiovascular diseases can contribute to dysbiosis in the gut microbiota. On the other hand, the gut microbiota can also impact the cardiovascular system via its metabolites [11, 65]. For example, enhanced populations of *Bacteroides/Prevotellain*, *Eubacterium rectale*, and *Fusobacterium prausnitzii*, in patient with heart failure, as well as less abundant *Coriobacteriaceae, Erysipelotrichaceae*, and *Ruminococcaceae* [66, 67]. More recently, the notion of the gut-organ axis, such as the gut-brain axis [68], gut-heart axis [69], and gut-artery axis [70], has gained attention. This concept provides a solid foundation for understanding the relationship between the gut microbiota and multiple cardiovascular diseases [54, 71–73].

   SCFAs, such as acetate, butyrate and propionate, are small molecular metabolites produced through the fermentation of dietary fiber. They perform a crucial function in the regulation of immunological equilibrium, bolstering the integrity of the intestinal barrier, and serving as a source of energy for the host [74–76]. Study has shown that SCFAs, particularly butyrate, can support the failing heart by bypassing the enzyme CPT1 and serving as an alternative energy source [15]. Importantly, a decrease in SCFAs due to gut dysbiosis can disrupt the integrity of the intestinal barrier, resulting in the translocation of microbial metabolites and inflammation, thereby promoting the development of cardiovascular diseases. For instance, a cohort study identified that dysbiosis and reduced butyrate production were associated with the aggravation of abdominal aortic aneurysm (AAA) [77]. Another study found that propionate could alleviate vascular calcification induced by vitamin D3 deficiency by restoring the homeostasis of intestinal microorganisms, promoting SCFA production, preserving intestinal integrity, preventing “leakage,” and inhibiting inflammation [78]. Moreover, gut dysbiosis, characterized by reduced butyrate production, can increase intestinal permeability and systemic inflammation, which in turn promote atherosclerosis and heart failure.

   Another metabolite, called trimethylamine N-oxide (TMAO), has also been found closely linked to cardiovascular disease. Research conducted by Makrecka-Kuka et al. demonstrated that feeding mice with 120 mg/kg of TMAO for 8 weeks resulted in elevated plasma TMAO levels, which subsequently impacted cardiac energy metabolism and mitochondrial function. This influence on energy metabolism and mitochondrial function ultimately led to ventricular remodeling and the development of heart failure [79]. Furthermore, Savi et al. discovered that TMAO affected the contractile function and intracellular calcium processing in cardiomyocytes. This effect is believed to be due to reduced energy production caused by TMAO-induced mitochondrial dysfunction [80]. In addition to these findings, studies have shown that the gut microbiota can influence platelet hyperresponsiveness and the formation of blood clots through the production of TMAO [81].

   In addition to TMAO and SCFAs, recent investigation has revealed the involvement of a gut microbiota-derived metabolite called Trimethyl-5-aminovaleric acid (TMAVA) in cardiovascular health. This study suggests that TMAVA can reduce fatty acid oxidation (FAO) and increase lipid accumulation, leading to the inhibition of carnitine synthesis and uptake. As a result, it can accelerate cardiac hypertrophy, a condition characterized by an abnormal increase in the size and mass of the heart [82]. This dysbiosis, or imbalance in the gut microbiota composition, is thought to be associated with increased inflammation and oxidative stress, which can further contribute to the progression of acute heart failure. While there is growing evidence suggesting a role of dysbiosis in the pathogenesis of DIC, the definitive role of gut microbiota dysbiosis in this condition remains unclear. Additional investigation is required in order to have a comprehensive understanding of the causes and consequences of gut microbiota-derived metabolites in relation to cardiovascular diseases and their association with DIC.

### The potential pathogenesis involved in gut microbiota in DIC

   Drugs also have significant effects on the composition and function of intestinal microbiota [83]. The interactions between drugs and microbiota can influence bacterial metabolism, as well as the activity and effectiveness of the drugs [84]. Previous studies have found that doxorubicin can cause substantial changes in the intestinal flora [85, 86]. Several studies have shown that mice treated with doxorubicin exhibited gut dysbiosis compared to the control group [87–89]. For instance, certain bacteria like *Faecalibaculum*, *Dubosiella*, and *Lachnospiraceae* were found to be increased in mice treated with DOX, while others such as *Allobaculum*, *Muribaculum*, and *Lachnoclostridium* were decreased [88]. Additionally, a recent study indicated that doxorubicin could reduce its toxicity to a model species called *Caenorhabditis elegans* through the presence of *Raoultella panticola* [90]. Notably, *Lactic acid bacteria (LAB)* and *Bifidobacteria* play an important role in the gut microbiota balance of the host health. However, study showed that most *LAB* and *Bifidobacteria* were easily inhibited by DOX [85]. Lin and colleagues showed that DOX treatment significantly altered the gut microbiota composition in rats, leading to a reduction in *Bacteroidetes* and *Verrucomicrobia*, and an increase *Proteobacteria*, and *Epsilonbacteraeota* [91]. Further investigations revealed that this dysbiosis was associated with cardiac inflammation and mitochondrial dysfunction, contributing to the development of cardiotoxicity. Notably, the transplantation of fecal microbiota from DOX-treated mice to germ-free (GF) mice induced cardiotoxicity, indicating a key role of gut microbiota dysbiosis in DIC [87]. Moreover, it was shown that *Proteobacteria* exhibited a considerably high prevalence in rats treated with DOX, suggesting that it might possibly serve as a sensitive indicator to distinguish between rats with and without cardiotoxicity [91]. With the advancement of metabolomics and 16s rDNA gene sequencing technique nowadays, more biomarkers may be used as a diagnostic in diseases. For instance, recent study has shown that an increased abundance of *Proteobacteria* is a potential diagnostic signature for dysbiosis and disease risk [92]. Similarly, a multicenter clinical trial indicated that special microbiota (e.g. *Enterococcus spp*) can be used as potential biomarkers for patient to monitor during intensive care unit stay [93]. Furthermore, a systematic review demonstrated that gut microbiota-generated metabolite TMAO was clearly associated with cardiovascular risk and mortality, and may be a potential biomarker [94]. Despite it remains challenging to identify specific gut microbiota dysbiosis in drug-induced cardiotoxicity, but it shows promise as a diagnostic tool.

   Although the gut microbiota dysbiosis caused by DOX has been reported, the exact mechanism by which this dysbiosis affects drug-induced cardiotoxicity is not fully understood. A more comprehensive understanding of this relationship could potentially contribute to the development of effective cardioprotective adjuvants. Currently, several possible mechanisms have been proposed, including intestinal barrier dysfunction, inflammatory response and immune activation, alteration of SCFAs and Hydrogen sulfide (H_2_S).

#### Intestinal barrier dysfunction

   The intestinal barrier is primarily made up of three components: the mucus layer, an epithelial barrier, and a gut vascular barrier. These components play a crucial role in maintaining overall health and preventing disease by facilitating nutrient absorption and simultaneously preventing the entry of pathogens [95]. Actually, the intestinal barrier is constantly exposed to various immunological and microbiological factors. When the barrier function is compromised, pathogens and bacterial metabolites such as lipopolysaccharides (LPS) can enter the bloodstream, leading to damage in distant organs, including the heart [96]. Previous studies in mice have indicated that the presence of gut microbiota can lead to intestinal mucosal damage when exposed to DOX [97]. These damages included the decrease in crypt number, crypt proliferation, and villus height. Besides, DOX administration also resulted in significant increases in apoptosis in jejunal epithelium. Immunostaining for MUC2 and lysozyme indicated the changes of goblet cells, Paneth cells or dual stained intermediate cells [97].

   The epithelial barrier function relies on the presence of a continuous layer of cells and tight junctions that seal the space between these cells, known as the paracellular space. The interecellular junctions consist of tight junction (zonula occludens), the adherens junction (zonula adherens), and the desmosome. Among them, the tight junction is the primary determinant of paracellular permeability. Molecularly, claudins are the most important of the transmembrane tight junction proteins [98]. The ZO family proteins, including ZO-1, ZO-2, and ZO-3, serve as scaffolding proteins that directly interact with transmembrane tight junction proteins like claudins and the tight junction-associated Marvel protein (TAMP) family, which includes occludin. These interactions help to regulate tight junction assembly and stability, thereby maintaining the integrity of the epithelial barrier [99]. A recent study has demonstrated that treatment with DOX can disrupt the organization of tight junction proteins, such as CLAUDIN-2, leading to an increase in the passage of small macromolecules, including bacterial products, across the epithelial barrier. This occurs due to alterations in the expression of tight junctional components and the loosening of cellular tight junctions, ultimately compromising the integrity of the barrier [100]. Consequently, the development of DIC has been linked to impaired intestinal barrier function. Moreover, a clinical study also found that increased gut permeability was associated with the deterioration of chronic heart failure [101]. Recently, abundant studies have taken the intestinal barrier as a potential therapeutic target in many diseases [102–105]. Regrettably, there is limited research available regarding the relationship between DIC and intestinal barrier homeostasis. Additional research is required to investigate whether targeting the intestinal barrier could be a promising therapeutic approach for the treatment of DIC.

#### Inflammatory response and immune activation

   Gut dysbiosis leads to the translocation of bacterial products (especially LPS) from the gut into the bloodstream, causing immune dyshomeostasis and systemic inflammation. A study by Zhao et al. showed that intestinal bacteria translocation influences myocardial ischemia/reperfusion (I/R) injury by amplifying the inflammatory response, and they proposed a novel concept known as the heart-gut-microbiome-immune axis [106]. Additionally, Tian et al. discovered obvious dysbiosis of gut microbiota and changes in metabolites in patients with AAA through the use of 16s rRNA gene sequencing and metabolomics. Further investigation in AAA mice demonstrated that *R. intestinalis* and its metabolite butyrate significantly reduce neutrophil infiltration and NOX2-dependent neutrophil extracellular trap formation, thus alleviating inflammation and markedly reducing AAA development [77]. These findings suggest that inflammation caused by gut microbial invasion and translocation play an important role in the progress of cardiovascular disease.

   Gut microbial dysbiosis and immune response activation can contribute to DIC [89]. For instance, several studies have shown that DOX can induce intestinal mucositis and damage [107]. As a result, LPS can enter the bloodstream through the compromised intestinal barrier, triggering the expression of various downstream inflammatory products such as tumor necrosis factor (TNF), IL-1, and IL-6, via the TLR4 pattern recognition receptor. Systemic inflammation, in turn, contributes to DIC by activating proinflammatory signaling pathways in the heart. Additionally, TMAO, a signaling molecule derived from bacterial species like *Firmicutes* and *Actinobacteria*, has been implicated in inflammatory modulation by promoting the release of IL-18 and IL-1β, and it has been closely associated with cardiovascular diseases such as atherosclerosis, hypertension, and heart failure [63, 108–110]. Zhang et al. reported that TMAO promoted vascular calcification through the activation of NLRP3 inflammasome and NF-κB signals [111]. Similarly, Li et al. demonstrated that TMAO exacerbates DOX-induced cardiac fibrosis by upregulating the expression of TLR4 and activating the NLRP3 inflammasome [112]. These findings indicate that alterations in gut microbiota may contribute to the development of doxorubicin-induced cardiomyopathy, involving interactions with the host immune system.

#### Alteration of SCFAs

   As mentioned before, SCFAs participate in anti-inflammatory responses and immunomodulation in the host. There is evidence suggesting that a decrease in SCFAs can lead to mitochondrial dysfunction, oxidative stress, and inflammation in the heart, contributing to DIC [18, 113, 114]. Chen et al. conducted a study that revealed a mouse model induced by programmed cell death 1(PD-1) or its ligand 1(PD-L1) inhibitors exhibited gut microbial dysbiosis. This dysbiosis was characterized by a notable decrease in SCFA-producing bacteria such as *Prevotellaceae* and *Rikenellaceae*, resulting in lower production of butyrate, an important SCFAs. The oral administration of butyrate-producing bacteria, such as *Prevotellaloescheii*, or butyrate itself has been shown to alleviate cardiotoxicity [115]. This effect was achieved by reducing the expression of pro-inflammatory cytokines like IL-1β and TNF-α and promoting the polarization of M2 macrophages in the colon. As a result, inflammatory responses were reduced, leading to a mitigation of cardiotoxicity.

   Similarly, a recent and promising study has demonstrated, for the first time, that oral administration of FAB, a novel synthetic derivative of butyrate, can protect the heart from DIC. This protective effect was mediated by reducing cardiac fibrosis and apoptosis, as well as decreasing levels of nitrosative and oxidative stress mediators, thereby improving mitochondrial dysfunction [18]. Recently, Nataly and colleagues have demonstrated that both butyrate and AN-7 (a butyric acid prodrug) could protect against DIC by reducing inflammatory factors [17]. Furthermore, oral administration of butyrate was shown to reduce cardiomyocyte apoptosis and induce an anti-inflammatory effect by promoting the polarization of anti-inflammatory M2 macrophages in the colon [89]. Another study found that butyrate could not only enhance the anticancer activity of doxorubicin but also protect cardiomyocytes from the side effects induced by DOX [16]. However, the specific mechanism of action still requires further exploration.

#### Alteration of hydrogen sulfide (H_2_S)

   Hydrogen sulfide (H_2_S) is an essential endogenous gaseous molecule that exert an important role in modulating numerous physiological and pathological processes, including cardiovascular diseases [116]. Studies have demonstrated that H_2_S can protect cardiomyocytes from DIC through its anti-inflammatory, anti-apoptotic, and antioxidant properties [117]. Moreover, emerging study suggests that H_2_S donors and prodrugs have dual activity, which not only synergizes with the anticancer effects of DOX but also protects cardiomyocytes from doxorubicin-induced damage [118–120]. These findings indicate that H_2_S holds great promise as a preclinical myocardial protective agent in the context of DIC.

   The majority of existing research has been concentrated on endogenously produced H_2_S by the host. However, it is worth noting that gut microbiota also produces H_2_S, which has the potential to impact human homeostasis. Some studies have reported a close association between gut microbiota-derived H_2_S and conditions like hypertension and atherosclerosis [121–123]. Furthermore, several studies have identified a correlation between the dysbiosis of gut microbiota in GF mice and the reduction of cystathionine gamma-lyase (CSE) activity in several organs, including the heart. This suggests that gut microbiota may also impact the expression of CSE in cardiomyocytes [124]. However, further investigation is needed to determine whether H_2_S derived from the gut microbiota is involved in the onset and progression of DIC.

**Fig. 2:**
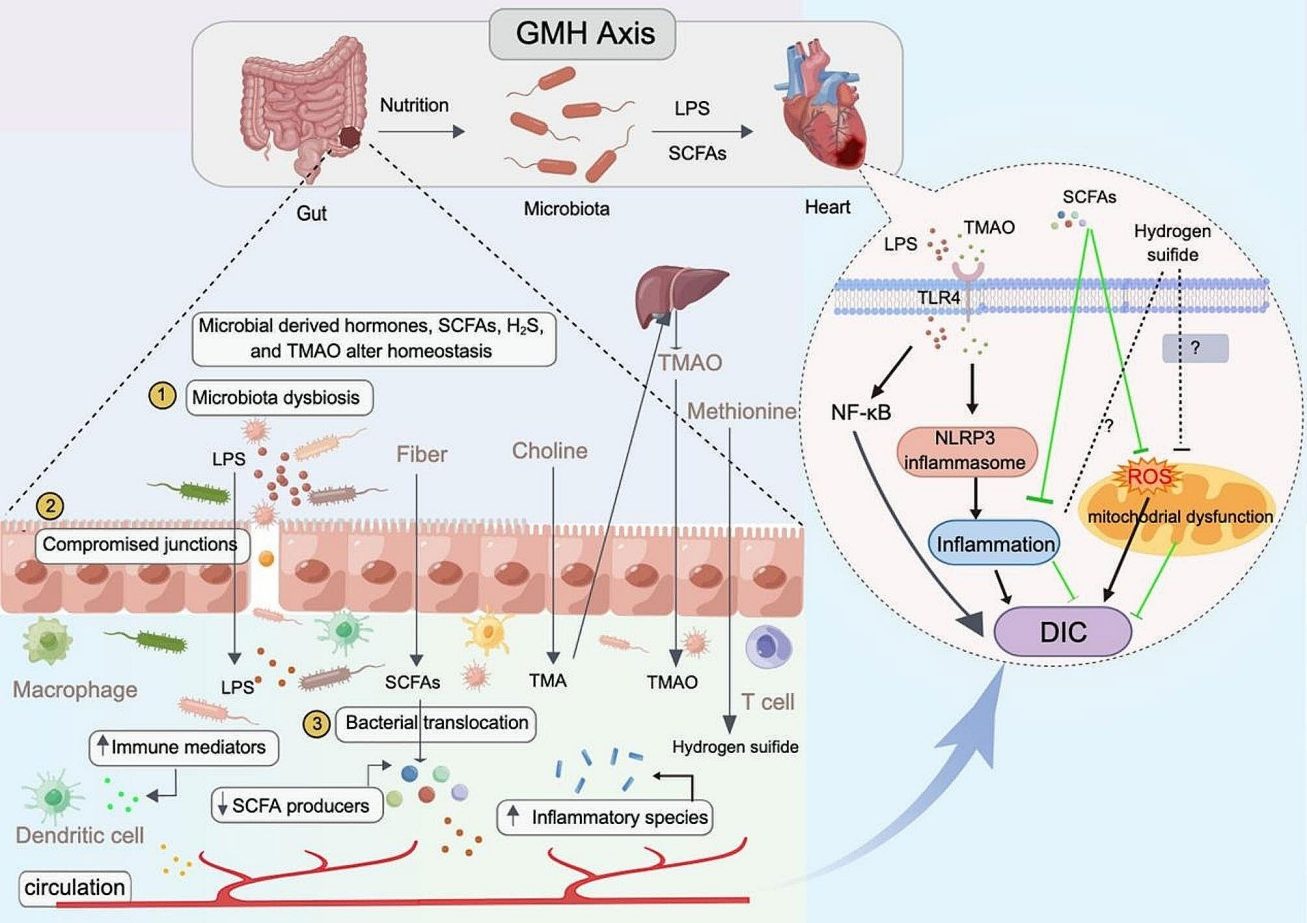
The role of gut microbiota and its metabolites in DIC. Gut microbiota dysbiosis destroys the tight junctions, leading to gut bacterial translocation, entry of bacterial components (such as LPS) into the circulatory system, and induction of chronic inflammation by harmful metabolites such as TMAO and pro-inflammatory factors, causing harm to the cardiovascular system via the gut-microbiota-heart axis; Metabolites (like LPS, TMAO) produced by gut microbiota can enter the bloodstream and promote to the TLR4-mediated production of a wide range of proinflammatory response pathway (e.g. NF-κB and NLRP3), thus aggravating the DIC. However, SCFAs play an anti-inflammatory role in this axis. *Abbreviations* SCFAs, short-chain fatty acids; ROS, reactive oxygen species; TMAO, trimethyl-amine N-oxide; H_2_S, hydrogen sulfide; LPS, lipopolysaccharides; The figure was created with Figdraw (https://www.figdraw.com/)

   In summary, gut microbiota dysbiosis appears to exacerbate the development of DIC, including inflammatory responses and immune regulation (Fig. 2). Although the precise mechanism linking gut microbiota and DIC remains incompletely understood, the aforementioned research suggests that targeting the gut microbiota could be an encouraging strategy for ameliorating DIC.

## The potential interventions of microbiota-targeted therapeutics in DIC

   The occurrence of DIC varies widely and is influenced by factors, such as lifetime cumulative dose and underlying comorbidities in patients. Evidence suggests that patients with risk factors like obesity, diabetes, and hyperlipidemia are more susceptible to DIC due to metabolic remodeling [48]. Recent studies have also demonstrated the beneficial role of gut microbiota in reducing cholesterol levels in the host, providing a strong foundation for the development of novel probiotics for the prevention and treatment of cardiovascular diseases [125]. Additionally, numerous animal studies and a limited number of human studies have shown promising potential of gut microbiota in preventing and treating cardiovascular diseases and metabolic syndrome, generating widespread interest among researchers [126–128]. While few studies have directly linked the gut microbiota to the risk factors associated with doxorubicin-induced cardiotoxicity and improving these risk factors to mitigate cardiotoxicity, this direction holds promise as a novel area for further investigation.

**Fig. 3:**
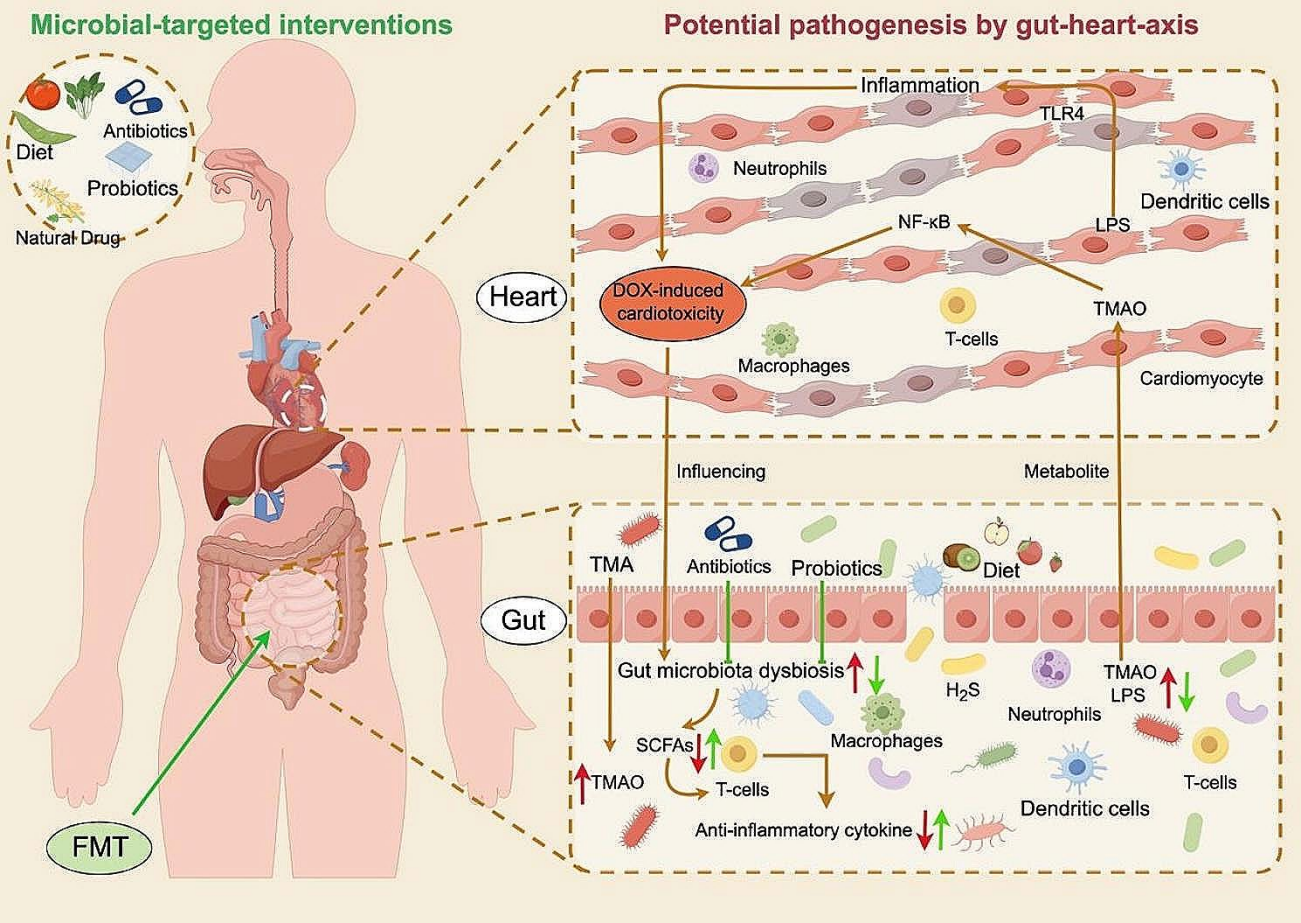
The potential role of the gut-microbiota-heart axis in the pathogenesis and microbial-targeted interventions in DIC. DIC led to gut microbiota dysbiosis, decreased SCFs, increased pro-inflammatory cytokines and LPS, activated inflammatory signaling pathways (e.g. TLR4 and NF-κb), aggravated DIC. Whereas, microbiota-targeted interventions could alleviate DIC through reverse the microbiota dysbiosis. Probiotics, diet, FMT, and natural phytochemicals alleviate the DIC via improving beneficial microbiota and producing SCFAs. SCFAs modulate immune cells (e.g. T-cells) to release anti-inflammatory cytokine reducing inflammation. Abbreviations: DOX, doxorubicin; YWPC, yellow wine polyphenolic compound; FMT, Fecal microbiota transplantation; ALPP, Arctium Lappa L; GLA, Glabridin; The figure was created with Figdraw (https://www.figdraw.com/)

In recent years, there has been an increasing scholarly focus on the interaction between microbiota and anticancer medications, along with the exploration of therapies aimed at shaping the microbiota to enhance therapeutic effectiveness and mitigate adverse reactions [129]. Numerous studies have demonstrated that gut microbiota not only influence chemotherapy effectiveness but also modulate the side effects of anticancer drugs, as previously reviewed [130]. A new field called Pharmacomicrobiomics has emerged to explore the impact of variations in microbiome composition and function on drug efficacy, toxicity, and pharmacokinetics [130]. The diversity of gut microbiota grants it an extraordinary metabolic capacity surpassing that of the host [131, 132]. Gut microbiota can produce a range of metabolic responses to drugs and xenobiotics, leading to both direct effects on drug metabolism and toxicity. These responses can ultimately influence individual variability in drug response [133]. Thus far, several enterobacteria capable of inactivating DOX, by deglycosylation to 7-deoxydoxorubicinol and 7-deoxydoxorubicinolone, have been identified, such as *Raoultella_planticola* [90]. Microbial detoxification of DOX could impact its therapeutic concentration in cancer patients, significantly mitigating its off-target toxicity [134]. In this context, the simultaneous administration of probiotics, symbiotics, postbiotics, or antibiotics with anticancer therapy has been proposed to restore balance to the gut microbiota [135]. For instance, Wang et al. indicated that cytotoxic T lymphocyte antigen-4 (CTLA-4) related-colitis was ameliorated by giving probiotic *Bifidobacterium* in mice [136]. Similarly, supplement of probiotics could reduce chemo/radiation therapy toxicity in many cancers’ treatment as well [137]. This section will describe the potential interventions of microbe-targeted therapeutics in DIC (Fig. 3).

### Dietary interventions

   Gut microbiota is closely linked to daily diets, and even a short-term adjustment in the diet, as short as 5 days, is sufficient to alter the composition of gut microbiota and induce corresponding changes for adaptation [138]. Stewart et al. discovered that long-term ingestion of milk or kefir (a milk containing lactic acid bacteria) could be beneficial for rats treated with doxorubicin [139]. Additionally, it has been demonstrated that the administration of phenylalanine-butyramide (a novel synthetic derivative of butyric acid) can have a positive effect on DIC [18]. A recent study has shown that supplementation of dietary Zn(ii)-curcumin (ZnCM) can attenuate gut dysbiosis during DOX-induced cardiomyopathy in vivo [140]. Furthermore, recent investigation has indicated that intermittent fasting (IF) has the potential to modulate the composition of gut microbiota, contributing to improvements in obesity and host energy metabolism [141]. Interestingly, a recent significant study found that alternate-day fasting (ADF) can aggravate DIC through transcription factor EB (TFEB)-mediated autophagy [41]. However, whether ADF affects the occurrence of DIC by altering gut microbiota is an area that deserves further exploration. Overall, dietary interventions have the potential to influence gut microbiota and assist in the prevention of DIC, but additional trials are needed to establish their efficacy.

### Probiotic therapy

   Probiotics are live microorganisms that provide health benefits to the host when administered in sufficient amounts [142]. They are the most extensively studied microbiota-targeting therapies for cardiovascular diseases [143]. Probiotics are known to alter the intestinal microbiota, create substances that can protect against harmful microbes, increase the integrity of the intestinal barrier, and reduce intestinal inflammation, however the exact mechanism by which they work is yet unknown [144]. Additionally, it was show that probiotics also had anti-oxidant and anti-inflammatory activities [145]. A study conducted by Zhao et al. has demonstrated that *Lactobacillus* supplementation can prevent cisplatin-induced cardiotoxicity possibly by inhibiting inflammation [146]. Similarly, recent animal studies have shown that the consumption of probiotics may have potent anti-cytotoxic effects [147], and a diet containing yogurt components can improve membrane integrity and cardiac contractility in a rat model of DOX-induced cardiomyopathy [148]. This probiotic treatment restored the composition and function of gut microbiota, reduced oxidative stress and inflammation, and improved DOX-induced cardiomyopathy [148]. Amaretti et al. observed an increase in reactive oxygen species metabolites in the plasma of rats treated with DOX, which was alleviated after supplementation with *Bifidobacterium* [149]. Based on the above research findings, supplementation with probiotics after DIC occurrence can alleviate myocardial cell damage and improve cardiac function. The application of probiotics to patients with DIC could serve as one of the auxiliary treatment options for clinical physicians. Additionally, probiotics may offer benefits by addressing microbiota dysbiosis-associated risk factors for DIC, such as obesity, aging, and hypertension. Although it has been reported that probiotics could protect against DIC, further work is needed to clarify the possible mechanisms and specific beneficial strains.

### Fecal microbiota transplantation

   Fecal microbiota transplantation (FMT) is a strategy to improve gut microbiota dysbiosis via also transferring feces from a healthy donor to the intestines of another patient [150]. Preclinical studies have demonstrated that FMT has encouraging outcomes in alleviating heart failure. Chen et al. recently demonstrated that recolonization with *Prevotella loescheii* and supplementation with butyrate reduced cardiotoxicity associated with PD-1/PD-L1 inhibitors [115]. Another study indicated that FMT improved DOX-induced cardiac dysfunction by altering the composition of the gut microbiota [87]. Whereas there have been reports on the adverse effects and complications of FMT therapy, such as the introduction of viral communities [151], this treatment approach is gaining momentum in scientific research and clinical practice. One promising development is the exploration of alternative forms of FMT, such as pills derived from human feces, which offer a less invasive and more standardized approach. It is imperative to acknowledge that further investigation is required so as to comprehensively understand the underlying benefits, risks, and optimal applications of FMT in the context of cardiotoxicity and other conditions.

### Natural phytochemicals

   Natural drugs primarily refer to pharmacologically active natural products found in nature, such as plants [152]. Increasing evidence suggests that natural phytochemicals have the ability to influence both gut microbiota and cardiac function [153]. Lin et al. conducted a study demonstrating that a polyphenolic compound from yellow wine (YWPC) effectively alleviated inflammation induced by DOX and improved mitochondrial function. This effect was attributed to the modulation of gut microbial ecology and related metabolites [91]. Similarly, another study showed that the flavonoid Glabridin (GLA) regulated DOX-induced gut microbiota dysbiosis, which contributed to the prevention of cardiotoxicity, potentially involving the *Desulfovibrio genus* [89]. Additionally, Wu et al. observed that polyphenols from Arctium Lappa L. (ALPP) improved gut microbiota dysbiosis and ameliorated heart failure in mice treated with doxorubicin. ALPP increased the abundance of *Lactobacillaceae*, *Muribaculaceae*, and *Ruminococcaceae*, while decreasing the abundance of *Proteobacteria*, *Enterobacteriaceae*, and the *Escherichia*_*Shigella* group compared to the DOX-treated group [154]. These results demonstrate the potential of natural compounds in modulating the gut microbiota and mitigating cardiotoxicity. However, to completely understand the potential mechanisms of action and to maximize their therapeutic application, more study is necessary.

### Antibiotics

   Antibiotics(Abs) possess both positive and negative effects [155]. The improper utilization of antibiotics has the potential to disturb an individual’s microbiome, resulting in adverse outcomes. Nevertheless, in specific circumstances, antibiotics can prove advantageous, particularly when adverse events are triggered by microbial translocation. One example is the utilization of Rifaximin as a therapeutic intervention for the management of microbiota toxicity and translocation. This medication has anti-inflammatory properties and facilitates the proliferation of advantageous microbial species, such as *Bifidobacteria* and *Lactobacillus* [156]. Similarly, a study by Shen et al. showed that mechanical hyperalgesia induced by oxaliplatin was alleviated in germ-free (GF) mice and mice treated with antibiotics, compared to the control group [157]. Huang et al. conducted an experiment in which they depleted the gut microbiota using antibiotics and found that this intervention could alleviate DIC [88]. Furthermore, a recent study demonstrated that minocycline could improve DIC by reducing inflammation and oxidative stress [158]. However, some studies have indicated that Abs-induced gut microbiota dysbiosis exacerbates the disease [159, 160]. For example, recent findings demonstrated that mice treated with antibiotics before myocardial infarction (MI) exhibited significantly increased mortality compared to MI mice models not administered antibiotics. Further investigation revealed an association between MI and gut microbiota dysbiosis, particularly affecting *Lactobacillus* [161]. Additionally, a rat study showed that broad-spectrum antibiotic treatment worsened DIC. Subsequent 16s rDNA gene sequencing revealed significantly higher abundance of *Klebsiella*, *Providencia*, and *Parasutterella* in the DOX + Abs group, whereas the abundance of *Muribaculaceae_unclassified*, *Lactobacillus*, and *Firmicutes_unclassified* was significantly lower compared to the DOX group [91]. While there is limited research on antibiotics-based treatments for DIC, antibiotics show promise as a microbial therapeutic approach for this condition. Notably, before considering antibiotics as a viable therapeutic option, further research is necessary to elucidate their precise mechanisms of action on gut microbiota and potential benefits in DIC. Otherwise, indiscriminate use may result in nonspecific depletion of the microbiota. The representative study of gut microbiota in the treatment of DIC is summarized in Table 1.

**Table 1:** Representative research on gut microbiota for the treatment of DIC

Interventions	Model	Microbiota changes	Main findings	Ref
FBA	Mice	N/A	Reduced oxidation stress, mitochondrial dysfunction.	[18]
FMT	Mice	↑*Prevotellaceae_UGG-001*, *Alloprevotella*, *Rilennellaceae_RC9*	Increased ZO-1 expression, improved cardiac function, altering microbiota composition.	[87]
Antibiotics	Mice	Depletion of gut microbiota	Alleviated DOX-induced myocardial injury and cardiomyocyte apoptosis.	[88]
GLA	Mice	↑*Lactobacillus genus* ↓*Desulfovibrio genus*	Prevented DIC, altered gut microbiota and colonic macrophage phenotype.	[89]
YWPC	Rat	↑*Alloprevotella, Eubacteriaceae*, *Negativibacillus;* ↓*Proteobacteria*, *Escherichia-Shigellagroup, Gammaproteobacteria*	Alleviated inflammation and mitochondrial dysfunction, modulated gut microbial community;	[91]
ZnCM	Rat	↑*Firmicutes* ↓*Bacteroidetes*	Alleviated gut dysbiosis, improved heart function; reduced cardiomyocyte apoptosis;	[140]
ALPP	Mice	↑*Lactobacillaceae, Muribaculaceae*, *Ruminococcaceeae*; ↓*Proteobacteria*, *Enterobacteriaee*, *Escherichia_Shigella*;	Reduced heart failure; improved gut microbiota composition;	[154]

*Abbreviations* DIC, doxorubicin-induced cardiotoxicity; SCFA, short chain fatty acid; FBA, phenylalanine-butyramide; YWPC, yellow wine polyphenolic compound; ZnCM, Zn(ii)-curcumin; FMT, Fecal microbiota transplantation; ALPP, Arctium Lappa L; GLA, Glabridin; N/A, not applicable

## Conclusions and future perspectives

   The gut microbiota exerts a crucial role in the regulation of cardiovascular diseases, and emerging study suggests that changes in the gut microbiota and its metabolites may potentially contribute to the onset of DIC. Targeting the gut microbiota presents a potential avenue for novel DIC prevention strategies. Currently, various microbial-targeted interventions, such as dietary changes, FMT, probiotics, antibiotics, and natural phytochemicals, have been explored for DIC prevention. However, several important challenges need to be addressed to further advance the field:

(1) Mechanistic understanding: The extent and specific mechanisms by which alterations in the gut microbiota led to DIC are still not fully understood. (2) Establishing causal relationships: Determining the causal relationship between the gut microbiota and DIC poses a challenge that requires further investigation. (3) Identifying probiotic candidates: Identifying specific microbiota strains that can be considered as probiotics for DIC treatment is an ongoing research area. (4) Translating findings to clinical practice: While most studies on gut microbiota and DIC are conducted on animal models, it is crucial to achieve the translation of these findings to human clinical settings considering the differences between human and animal gut microbiota.

   Addressing these challenges will be essential for the development of effective microbial-based therapeutics for DIC prevention and treatment. To address these issues, several steps can be taken. Firstly, it is crucial to enhance interdisciplinary collaboration and carry out large-scale prospective studies to gather more comprehensive information. This can facilitate a better understanding of the relationship between gut microbiota and DIC. Secondly, more preclinical and clinical studies are needed to employ advanced techniques like high-throughput sequencing and multi-omics approaches. These methods can help identify the specific pathogenic and beneficial strains associated with DIC. Thirdly, the utilization of innovative tools such as engineered strains and isotope tracing can shed light on the underlying pathways involving bacteria and their metabolites.

   Currently, there is a lack of studies in this area, thus further research can be conducted from two perspectives: (1) Elucidating the functional roles of specific bacterial strains and their metabolites in the development and prevention of DIC. (2) Investigating the clinical translation of FMT from animal models to human patients, considering the differences in gut microbiota between species. By addressing these aspects, we can make significant progress in understanding the role of gut microbiota in DIC and developing effective microbiota-based therapies for the condition.

   In addition to the previously mentioned approaches, there are emerging strategies targeting energy sources and metabolic pathways that have shown potential benefits in DIC. These include intermittent fasting [41], ketogenic diet [162], and exercise [163]. These approaches have been found to have positive effects on DIC management. It is important to note that the gut microbiota is highly susceptible to external factors, such as diet. The composition of gut microbiota can vary significantly between individuals, even among healthy individuals. Therefore, future studies should aim to investigate and clarify the effects of various confounding factors on gut microbiota.

   The emerging field of pharmacomicrobiomics is paving the way for innovative therapeutic approaches to various diseases, including cardiovascular disease [133, 164]. This approach involves initially reviewing the gut microbiota profile before treatment and then analyzing the patient’s microbiota after treatment. Moreover, exploring the potential for personalized treatment based on each individual’s microbiota and their ability to metabolize drugs correctly holds great promise. It is noteworthy that some patients with specific microbiota diversity may not metabolize drugs like DOX correctly, leading to potential harm instead of a beneficial intervention.

   Understanding the impact of external factors on gut microbiota composition can provide valuable insights into the potential influence of dietary interventions, such as intermittent fasting and ketogenic diet, on DIC prevention and treatment. Additionally, exploring the role of exercise in modulating gut microbiota and its potential benefits in DIC management can contribute to the development of comprehensive treatment strategies. Further studies focusing on these areas will help unravel the intricacies of the gut microbiota-DIC relationship and pave the way for personalized interventions targeting the gut microbiota for DIC prevention and management.

## Data Availability

No data was used for the research described in the article.

## Abbreviations

AAA: Abdominal aortic aneurysm
Abs: Antibiotics
AKT: protein kinase B
ATP: adenosine triphosphate
AMPK: AMP-activated protein kinase
CTLA-4: cytotoxic T lymphocyte antigen-4
ETC: electron transport chain
FAB: phenylalanine-butyramide
FMT: Fecal microbiota transplantation
GF: germ-free
GLA: glabridin
H_2_S: hydrogen sulfide
IF: intermittent fasting
LPS: lipopolysaccharides
MI: myocardial infarction
NLRP3: nucleotide-binding domain-like receptor protein 3
NF-κB: nuclear factor-κB
OXPHOS: oxidative phosphorylation
PD-1: Programmed cell death 1
PD-L1: Programmed cell death ligand 1
PUFA: polyunsaturated fatty acids
ROS: reactive oxygen species
SCFAs: short-chain fatty acids
System Xc-: cystine-glutamate antiporter
TFEB: transcription factor EB
TMAVA: Trimethyl-5-aminovaleric acid
TMAO: trimethyl-amine N-oxide
TLRs: toll-like receptors
YWPC: yellow wine polyphenolic compound
ZnCM: Zn(ii)-curcumin
